# Spotted cotton oligonucleotide microarrays for gene expression analysis

**DOI:** 10.1186/1471-2164-8-81

**Published:** 2007-03-27

**Authors:** Joshua A Udall, Lex E Flagel, Foo Cheung, Andrew W Woodward, Ran Hovav, Ryan A Rapp, Jordan M Swanson, Jinsuk J Lee, Alan R Gingle, Dan Nettleton, Christopher D Town, Z Jeffrey Chen, Jonathan F Wendel

**Affiliations:** 1 Department of Plant and Animal Sciences, Brigham Young University, Provo, UT, 84062, USA; 2 Department of Ecology, Evolution, and Organismal Biology, Iowa State University, Ames, IA, 50011, USA; 3 The Institute for Genomic Research, A Division of the J. Craig Venter Institute, 9712 Medical Center Drive, Rockville MD 20850 USA; 4 Section of Molecular Cell and Developmental Biology and Institute for Cellular and Molecular Biology, University of Texas, Austin, TX, 78712, USA; 5 Center for Applied Genetic Technologies, University of Georgia, Athens, Georgia, 30602, USA; 6 Department of Statistics, Iowa State University, Ames, IA, 50011, USA

## Abstract

**Background:**

Microarrays offer a powerful tool for diverse applications plant biology and crop improvement. Recently, two comprehensive assemblies of cotton ESTs were constructed based on three *Gossypium *species. Using these assemblies as templates, we describe the design and creation and of a publicly available oligonucleotide array for cotton, useful for all four of the cultivated species.

**Results:**

Synthetic oligonucleotide probes were generated from exemplar sequences of a global assembly of 211,397 cotton ESTs derived from >50 different cDNA libraries representing many different tissue types and tissue treatments. A total of 22,787 oligonucleotide probes are included on the arrays, optimized to target the diversity of the transcriptome and previously studied cotton genes, transcription factors, and genes with homology to *Arabidopsis*. A small portion of the oligonucleotides target unidentified protein coding sequences, thereby providing an element of gene discovery. Because many oligonucleotides were based on ESTs from fiber-specific cDNA libraries, the microarray has direct application for analysis of the fiber transcriptome. To illustrate the utility of the microarray, we hybridized labeled bud and leaf cDNAs from *G. hirsutum *and demonstrate technical consistency of results.

**Conclusion:**

The cotton oligonucleotide microarray provides a reproducible platform for transcription profiling in cotton, and is made publicly available through http://cottonevolution.info.

## Background

*Gossypium *contains nine different genome groups comprising approximately 50 species whose phylogenetic relationships have been well-studied [1]. The A-, D-, and AD-genome groups have received special attention, as four different species [*Gossypium herbaceum *(A1), *Gossypium arboreum *(A2), *Gossypium hirsutum *(AD1) and *Gossypium barbadense *(AD2)] have been domesticated for their abundant seed trichomes. These species collectively provide the foundation for the textile industry worldwide, with most cotton today deriving from *G. hirsutum*, or upland cotton. Relationships among genome groups have been quantified in several studies, and the closest living relatives of the diploid genome donors to allopolyploid cotton have been identified [1-5]. The diploid donor of the allopolyploid A_T _genome [where the T subscript indicates the A genome in the tetraploid (AD) nucleus], was a species much like modern *G. arboreum *or *G. herbaceum*, whereas the allopolyploid D_T _genome is derived from a progenitor similar to modern *G. raimondii*. These well-established relationships provide a phylogenetic framework to investigate the evolution of gene expression both in terms of domesticated fiber production and polyploidy.

Microarrays are a powerful method to simultaneously measure relative expression levels for thousands of genes and they may be composed of cDNA inserts, short oligonucleotides, or long oligonucleotides. The advantages and disadvantages of each of these probe types have been extensively reviewed [6-8] We chose to create a long oligonucleotide microarray for cotton because of its low manufacturing cost, flexibility in design, homogeneous melting temperatures (T_m_), and relative ease of adding probes. A small EST assembly (~45,000 ESTs) was previously used to generate oligonucleotide probes for cotton fiber [9]. A larger scale EST assembly (> 150,000 ESTs) was recently produced as a community-wide effort by cotton researchers [10]. Subsequent additions of cotton ESTs to Genbank (> 210,000 ESTs) have been compiled into a large EST assembly [TIGR Cotton Gene Index 8, (CGI8)] [11]. These two assemblies constitute nearly all of the known genic sequence from cotton. Similar large-scale EST assemblies have been successfully used to design oligonucleotide microarrays for functional genomics investigations in model plants (*e.g. Arabidopsis*[12] and rice[13]) and non-model plants (*e.g*. maize[14] and tomato[15]).

Here we describe the design and creation and of a publicly available oligonucleotide microarray for cotton. Synthetic oligonucleotide probes were generated from the sequences of two different assemblies of cotton ESTs representing more than 50 different cDNA libraries and many different tissue types and tissue treatments [10,11] To illustrate their utility on printed microarray slides, we hybridized labeled bud and leaf cDNAs from *G. hirsutum*, and demonstrate technical consistency of results. As many of these EST sequences were derived from a fiber-specific cDNA library, this array also has direct application for analysis of the fiber transcriptome.

## Results and discussion

### Microarray design

We created an oligonucleotide microarray for cotton using fiber genes in Genbank, a reported EST assembly of >150,000 ESTs [10], and a recent assembly of >210,000 ESTs [11] as templates for probe design. From these sequences, we designed three sets of oligonucleotide probes (1,154, 12,006, and 9,629, respectively) and included all three sets (22,787 total oligonucleotides) on a single, publicly available microarray [16]. The first set of 1,154 oligonucleotide probes was designed from Cotton ESTs with homology to *Arabidopsis *genes with roles as regulators of chromatin, transcription, cell wall biosynthesis, and cell cycle [17].

The second set of oligonucleotides probes was designed from an exemplar sequence set [10] using Picky v1.0 [18]. An exemplar sequence refers to an example gene (*i.e*., the longest) chosen from a clustered set of unigenes by single-linkage clustering with BLASTN [10]. Picky prioritized the unique sequence of the identified oligonucleotide probes while maintaining a uniform probe-target melting temperature. 12,006 oligonucleotides (66 bp average length; 3.5 s.d.) with a relatively small range of melting temperatures (T_m_, 78.33 ± 1.40 s.d.) were selected from a large list of candidate probes. This list of targeted genes includes genes requested by members of cotton research community, a large number of transcription factors, and several thousand genes that had homology to *Arabidopsis *genes (Table 1) [see Additional file 1].

**Table 1:** Oligonucleotide probes were designed separately from three different sets of ESTs.

Types of probes	1^st ^oligo set^1^	2^nd ^oligo set^2^	3^rd ^oligo set^3^	Totals^4^
*Arabidopsis *matches^5^	866	7,419	4,031	12,316
Singletons	na	5,280	3,852	9,132
Transcription factors (TF)^6^	230	2,223	677	3,130
GO Biological Process^7^	46	464	126	636
GO Molecular Function^8^	184	1,759	551	2,494
PFAM^9^	na	471	na	471

Total number of oligos	1,154	12,006	9,629	22,789

^1^Oligonucleotides (oligos) were designed at Texas A&M University in the former lab of Dr. Chen [17]. ^2^Oligos designed at Iowa State University from a global assembly of ESTs [10]. ^3^Oligos designed by The Institute for Genomic Research (TIGR) from the Cotton Gene Index 8 [11]. ^4^Column totals are close approximations since each oligo set was designed separately. ^5^*Arabidopsis *matches are based on oligo WUBLASTX hits to the TAIR *Arabidopsis *protein dataset and parsed for 50 aa length and 50% percent identity. ^6^Transcription factor sub-total may be an over-estimate as Biological Processes and Molecular Function are not mutually exclusive categories. ^7^Biological Process = transcription. ^8^Molecular Function = nucleic acid binding activity, nucleotide binding activity, RNA and DNA binding activity, and transcription factor activity. ^9^Putative PFAM TF identified in the 2^nd ^oligo set were in addition to those annotated by gene ontology.

The third set ofoligonucleotides probes was designed from CGI8 [11] that contained 55,673 unique sequences using Picky v2.0. Where possible, identical Picky parameters were used to design the 3^rd ^oligonucleotide set as the 2^nd ^set. Probes that targeted the same genes as in the first two probe sets were excluded from further analysis. In total, 9,629 additional oligonucleotides probeswere generated (66 bp average length, 3.6 s.d.; 76.82 T_m_, s.d. 1.93) and added to the previous 2 probe sets.

Two essential considerations of microarray quality include the number of targeted genes and the broad utility of the microarray for specific tissues or treatments. Regarding the first consideration, the 22,778 genes described here include perhaps 46–60% of the total genic diversity, given that the total number of genes in the cotton genome may be approximately 40,000–50,000 [19,20], Indeed, 44% and 40% of the oligonucleotides were designed from singletons from the first [10] and second [11] assemblies, respectively. However, some probes were not completely target-specific, perhaps due to imperfect EST assemblies and due to separate sets of probes designed from different assemblies. Approximately 1,800 of potential non-specific homologies were identified in the recent Gene Index of 55,673 unigenes [11] using vmatch [21] with a ~95% sequence percent identity threshold. Nevertheless, most of the oligonucleotides had a single target, as designed [see Additional file 2].

Regarding the second consideration, these microarray probes have a broad utility for specific tissues or treatments. For example, a detailed analysis of the second probe set revealed that ~7,300 probes represented genes expressed in specific tissues or under specific conditions [see Additional file 3]. The number of specifically expressed genes was determined by summing the number of contigs mostly composed of ESTs from a single library (90%) and the corresponding singletons. In total, 56% of the 2^nd ^oligonucleotide set represent genes from a specific library; however, a large number of those genes (24%) are from the two *G. raimondii *libraries that were prepared from heterogeneous tissues and which were more deeply sampled that most of the other cotton cDNA libraries. More than 1,000 oligonucleotides appear to target genes found only in a 7–10 days post-anthesis fiber library [9], and 733 appear to target transcripts uniquely identified following cyclohexamide treatment of ovules [22]. These two considerations suggest that the oligonucleotides selected for the cotton oligonucleotide microarray have a broad diagnostic utility while potentially targeting tissue-specific transcripts expressed under a variety of conditions. The sequences and annotations of all the probes are publicly available via a web-based query [16] or by request.

### Microarray hybridizations

Many potential sources of error can have a large impact on microarray experiments, such as inconsistency among multiple RNA extractions, reverse transcription, RNA amplification, and labeling, as well as different levels of background noise for each microarray. Many of these sources of error can be resolved by appropriate experimental design [23,24]. and careful laboratory technique; however, the quality of the microarray must often be assumed and often is not under the control of the investigator. We investigated the oligonucleotide performance within the first version of the cotton microarray [Gene Expression Omnibus (GEO) database: GPL4305] containing the 1^st ^and 2^nd ^replicated spots printed for each probe in these two sets. We found a low level of within-microarray variation, reproducible 'self vs. self' hybridizations with bud tissue, and reproducible expression differences between bud and leaf treatments. The results from the first version of the cotton microarray suggest that the current version with 9,629 additional probes will provide an robust, reproducible platform for transcription profiling in cotton.

**Figure 1:**
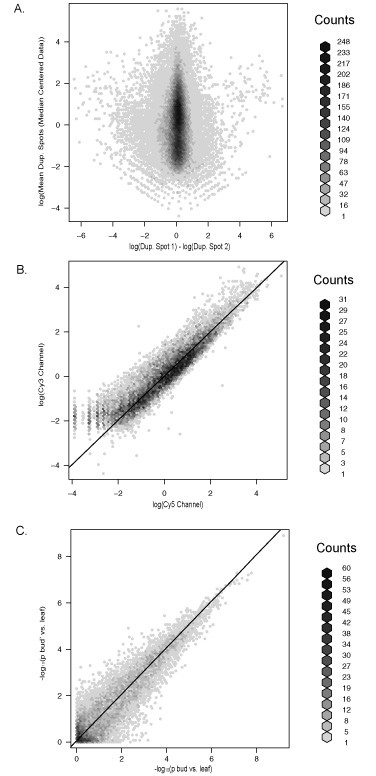
**A reproducible platform for transcription profiling using cotton microarrays**. A) Differences detected between duplicated spots plotted by their mean expression value from replicate 1 of the experiment. Each plot is hexagonally-binned to uncover the density component that is otherwise saturated by a cloud of data points. The difference of the log-adjusted, median centered duplicate spots is measured on the horizontal axis and the mean value of the same duplicated spots is measured on the vertical (values in grey, scale bars on left vertical axis). Most duplicated spots deviated very little, though genes with lower expression values tended to deviate more. Replication of the three treatment loop design indicated only minor detectable differences between duplicated spots. Nearly identical results were found for the other two replicates. B) Correlation of normalized, log-adjusted fluoresce intensity values for bud (Cy3) × bud' (Cy5) for the first microarray of the first replication. A 45° angle line has been overlaid to illustrate the expected 1:1 ratio of spot intensity. In this case, Cy3 labeled aRNA had higher intensity values on average; however, the effect of dye was removed from the contrast of differential expression in our analysis by including a dye component into our analytical model through dye swaps. C) Correlation of t-test p-values. Gene-specific tests for differential expression between bud and leaf and between bud' and leaf were conducted as described in Materials and Methods. The *p*-values from the bud' vs. leaf tests are plotted against the *p*-values from the bud vs. leaf tests on a negative natural log scale. The points in the upper right quadrant of the picture correspond to the genes with the smallest *p*-values. The points are scattered tightly around the 45 degree line, indicating that that *p*-values for the most significant genes were very similar according to both comparisons.

Variation within microarrays between the two replicate features was estimated for each oligonucleotide probe, as a consequence of including two microarray features for each oligonucleotide probe in two separate sections of the microarray. The average log-adjusted difference between two replicated features was 0.03 (s.d. 0.69), 0.02 (s.d. 0.80), and 0.02 (s.d. 0.83) for replications 1, 2, and 3, respectively (Figure 1A). The slightly positive value of the average spot differences suggests that the first pin-touch on the microarray deposited slightly more oligonucleotide probe in a slightly larger spot on the slide than the second pin-touch.

We also demonstrated the reproducibility of the microarray by quantifying the amount of variation for these newly constructed cotton microarrays between two identical pooled 'treatments' of bud RNA (bud and bud'). On the individual microarrays, there was a high degree of correlation between the Cy3-labeled bud RNA and the Cy5-labeled bud RNA (*r *= 0.92 data not shown). When searching for expression differences between bud and bud' RNA pools, no differences were detected at a relatively liberal false discovery rate of 20% (Table 2).

**Table 2:** Number of differentially expressed genes at different levels of false discovery [34].

Comparison	0.001	0.010	0.050	0.100	0.200	0.300
bud – bud'	0	0	0	0	0	20
bud – leaf	2167	4,506	6,562	7,600	8,933	9,694
bud' – leaf	2232	4,608	6,506	7,641	9,007	9,654

Finally, if we test for expression differences between the leaf RNA pool and each of the bud RNA pools (bud and bud'), many genes were found to be consistently differentially expressed between bud vs. leaf and bud' vs. leaf, as seen by the high correlation among the most significant p-values (Figure 1B). If our goal was to describe differentially expressed genes between leaf and bud, we would have a large list of putatively, differentially expressed genes (Table 2); however, in this particular case, we can only claim that these genes were differentially expressed between the two amplified RNA samples considered in our experiment. We intentionally did not include biological replicates because our interest was in quantifying technical variation. Biological replication would be necessary to conclude that the expression differences are inherent differences between leaf and bud rather than simply differences between the particular leaf RNA pool and the particular bud and bud' RNA pools prepared for this experiment.

Because a global assembly of ESTs was used to design this first version of the microarray, genes expressed in many tissues of the plants including cotton fibers, are represented. A total of 1,864 genes were found to be differentially expressed between our leaf RNA pool and our bud RNA pools (false discovery rate = 0.001; Figure 1C). (Again note that these are differences between our RNA pools rather than inherent expression differences between general leaf and bud tissues.) Of these differences, slightly less than 10% belonged to set of probes that had no BLASTX hit (< 1 × 10^-20^) and were designed solely based on an ESTScan prediction, thereby providing an element of functional gene discovery to the microarray. Of the 1,864 hypothetical genes that were differentially expressed, 602 were identified by probes derived from cotton fiber cDNA libraries [9]; Ben Burr, unpublished data; Candace Haigler, unpublished data). This number did not include probes designed for known cotton genes, nor other genes that were removed from the analysis (low expressed fiber genes that may not be detected in leaf or bud aRNA) that may also represent genes specifically expressed in fiber tissue.

## Conclusion

Here we provide a detailed report of the design of long-oligonucleotide microarrays for cotton and illustrate their technical performance. Proper design of microarray experiments for discovery of gene expression profiles requires biological replication [24-26] Because our goal was not to discover nor report novel gene expression profiles in leaves or buds, we restricted our replications to pools of technical replicates in this experiment. These cotton microarrays are publicly available [16], and may continue to be augmented with additional oligonucleotides designed from subsequent ESTs assemblies. As a tool for functional genomics, the future use of these microarrays may uncover clues to the transcriptional regulation of cotton fiber and other tissues in properly replicated experimental designs.

## Methods

### Microarray probe selection

A total of 22,787 probes were printed on the cotton oligonucleotide microarray, composed of 12,006 oligonucleotides selected from recently reported contigs and singletons derived from a global assembly of cotton ESTs [10], and 9,629 designed against the latest TIGR cotton transcript assembly (CGI8). We also included 1,154 oligonucleotides designed in the Z. J. Chen laboratory based on previously available sequence data [27].

Similar probe design strategies were used for all threes sets of probes. Lee et al. [17] provided a description for the design first set of oligonucleotides. The second and third set of oligonucleotides were designed from the EST exemplar sequences [10] and the TIGR Gene Index [11] with Picky (v 1.0) [18] and Picky (2.0), respectively. Only EST sequences with predicted a protein from ESTScan [28] or a high protein homology (70% percent identity) to an *Arabidopsis *gene were considered candidates for oligonucleotide selection.

Three different criteria were invoked in the iterative selection of the microarray probes from the EST assemblies: (1) candidate probes identified by Picky by their unique sequences, complexity, and T_m_; (2) characterized cotton mRNAs in GenBank and genes of special interest to the cotton community; and (3) complementation to previously synthesized probe set(s). Thus, for each oligonucleotide set, candidate long-oligonucleotide probes (60 – 70 mers) were separately generated based on criteria 1 and 2, then a final list of probes was selected for oligonucleotide synthesis by cross-checking the new list of probes with previously synthesized oligonucleotides.

For design of both the second and third probe sets, we solicited input from the cotton community to identify genes of interest for microarray probe design. Most requests were for known cotton genes with sequences in Genbank, in addition to candidate genes identified in our EST assembly by high homology to genes characterized in other organisms. Probes for these genes were designed with the sequences from the EST assemblies as 'background' to identify the most unique probes possible.

Both transcription factors and genes with little or no annotation were represented on the cotton microarray. Based on widespread interest in transcription factor expression levels, we selected probes targeting genes that had either a transcription related GO ontology [29], or a transcript factor domain as predicted by PFAM [30] (Table 1). Genes with little or no annotation represent a gene discovery component to future microarray experiments. When genes targeted by the 2^nd ^probe second were compared to the *Arabidopsis *TAIR protein dataset, 1,200 of them did not have a significant BLASTX hit (< 1e-20) but they did have a coding frame as predicted by ESTScan [28].

### Oligonucleotide synthesis and microarray printing

Each set of oligonucleotides was synthesized and aliquoted into 3 replicate plates by IDT Technologies (Coralville, IA, USA). An aliquot of 384-well plates from all three sets of oligonucleotides was hydrated in water then diluted to the printing concentration with 3× SSC. Positive and negative controls were included on the printed microarrays. To assess microarray quality, two spots of each oligo from the same pin-dip were printed in separate slide sections on Corning epoxy slides at the Washington University Microarray Core facility using a locally constructed linear servo arrayer (after the DeRisi model [31]) creating the first version of the cotton oligonucleotide microarray [GEO: GPL4305]. After printing, slides were allowed to dry in 50–70% humidity for 12–16 hrs (~25°C) and cross-linked at 150 mJoules. Two slides from the print batch were checked using SpotCheck (Genetix). Printed cotton microarrays, and images of each print batch are publicly available [16]. Experiments using the preliminary platform [GEO: GPL4305] or this new platform [GEO: GPL4808] can be found at GEO [32].

### RNA extraction

One leaf and one bud (10 – 14 days before anthesis) tissue sample of *G. hirsutum *cv. Acala Maxxa were collected from three separate replications of 4 – 8 plants grown in Horticulture Greenhouse at Iowa State University under supplemental lighting (16 hr. days). RNA was extracted from each of the six samples using a modified hot-borate method [33], quantified, and checked for integrity using a Bioanalyzer (Agilent, Inc., Palo Alto, CA, USA). Equimolar amounts of RNA (A_260_) from three separate extractions were pooled into a single leaf and single bud sample, respectively.

### RNA amplificationand labeling

An indirect labeling procedure of amplified aminoallyl.aRNA (TargetAmp™, Epicentre Biotechnologies, Madison, WI, USA) was used for one leaf RNA sample and one bud RNA sample. 0.5 ug of total RNA was used as starting material for 1 round of aRNA amplification, resulting in 26 ug and 51 ug of aRNA from leaves and buds, respectively.

Cy3 and Cy5 dyes (Amersham Biosciences, Pittsburgh, PA, USA) were coupled to two aliquots of 13 and 16 ug of both aRNA samples, respectively. The Cy3- and Cy5-labeled aRNA probes were purified using the Qiagen RNA easy Mini kit (Qiagen, Germantown MD, USA). and sufficient incorporation Cy3 (550 nm) and Cye5 (650 nm) dyes was verified.

### Microarray hybridization and image analysis

For microarray hybridization, 300 ng of Cy3 and Cy5 labeled aRNA was used per each slide using the Pronto!™ Plus system protocol (Promega Corporation, Madison WI, USA) with minor changes as described below. Slides from each rep (3) were immersed in 200 ml of Pronto Universal Pre-Soak solution containing 2 ml of liquid Sodium Borohydride for 20 min at 42°C. Slides were transferred to fresh containers with Wash Solution 2 at room temperature for 2 min and then immersed in 200 ml of hybridization buffer (5 × SSC; 0.1 × SDS; BSA 0.1 mg/ml). Slides then were incubated with a fresh Wash Solution 2 at room temperature for 2 min, and were washed 2 additional times with Wash Solution 3 at room temperature for 2 min each. Following immersion in nuclease-free water, slides were dried by centrifugation at 1,600 g for 3 min. All hybridizations and post-hybridization washes were performed exactly as described in the Pronto!™ Plus system protocol.

Microarray images were captured using an arrayWoRx^® ^Biochip Reader (Applied Precision, Issaquah, WA, USA) using an exposure of 0.5 sec for each channel (Cy5 and Cy3) at ~10 um resolution. GenPix^® ^Pro (v 5.1, Molecular Devices, Sunnyvale, CA, USA) was used to extract the background-adjusted intensity of each spot. Features that were 'absent', 'not-found', or that had a negative intensity after background adjustment were excluded from the analysis. Data files from this experiment can be found in GEO data set [GSE5875].

### Experimental design and statistical analysis

Three replications of a three treatment loop design (bud → leaf, leaf → bud', and bud' → bud) were hybridized on nine microarrays, where bud and bud' simply represent different aliquots of the same aRNA. The signal intensity data were natural log transformed and median normalized, and the 9,654 genes with complete data were examined for expression differences among the three sample types (leaf, bud, and bud'). We considered a standard mixed linear model for the data from any single gene given by

*y*_*ijk *_= *μ *+ *δ*_*i *_+ *τ*_*j *_+ *s*_*k *_+ *e*_*ijk*_,

where *y*_*ijk *_denotes the normalized log-scale signal intensity (averaged over duplicate spots) for dye *i*, sample type *j*, and slide *k*; *μ *denotes a an intercept parameter; *δ*_*i *_denotes the effect of dye *i*; *τ*_*j *_denotes the effect of sample type *j*; *s*_*k *_denotes the random effect of slide *k*; and *e*_*ijk *_denotes a random error term that is intended to capture all other sources of variability. (Note that although we considered a separate model for each gene, we have suppressed a gene subscript on each term to simplify notation.) Here *i *= 1, 2 (Cy3 and Cy5); *j *= 1, 2, 3 (bud, bud', and leaf); and *k *= 1, ..., 9 (microarray slides 1 – 9). On the basis of this model, *t*-tests for differential expression between each pair of sample types (leaf vs. bud, leaf vs. bud', and bud vs. bud') were conducted. The 9,654 *p*-values from each of these comparisons were converted to *q*-values using the method of Story and Tibshirani [34]. These *q*-values were used to identify the number of differentially expressed genes for a given comparison when controlling the false discovery rate at various levels.

## Abbreviations

ESTs: Expressed sequence tags

## Authors' contributions

JJW, JJL, and ZJC designed and provided the first set of oligonucleotides. JAU, JMS, JFW, and ZJC designed, analyzed, and provided the second set of oligonucleotides. FC, AWW, CDT, and ZJC designed and provided the final set of oligonucleotides. LF and JAU grew the plants and extracted RNA. RH, LF, and RAR amplified, labeled, and performed the microarray hybridizations. DN provided the statistical model and analytical framework. LF, JAU, and DN analyzed and interpreted the data. ARG customized an internet accessible MIAME-compliant Stanford Microarray Database for these arrays and created a GEO microarray platform for this and future experiment submissions. JAU, RAR, and JFW conceived the experiment. JAU and JFW drafted the manuscript. All authors have read and approved the manuscript.

## Availability and requirements

Project name: The evolutionary genomics of cotton

Project home page: http://cottonevolution.info

Operating system: Platform independent

Programming language: HTML and XML

License: no license required

## Supplementary Material

Additional file 1 **Composition of the cotton oligonucleotide microarray**. 22,789 oligonucleotides were designed from three separate sets of genic sequences from cotton (See Table 1). The grey boxes represent the total number of probes in each set. The hatched boxes indicate the number of probes with a putative *Arabidopsis *hit. The black boxes indicate the number of probes designed from singletons from their respective assemblies. The remaining boxes with dotted squares indicate the number of probes targeting transcription factors. Click here for file

Additional file 2 **Distribution of the number of matches of oligonucleotide probes to the Cotton Gene Index 8 (CGI8) assembly**. All three sets were queried within the sequences of the CGI8 assembly and only a small number (1,773) of probes target (>93% percent identity) more than one CGI8 unigene indicating a potential cross-hybridization or an 'over-split' assembly for the targeted gene. Click here for file

Additional file 3 **Many of the oligonucleotides from the 2^nd ^set were derived from contigs or singletons representing individual libraries (n = 7,319)**. Library totals reflect the contigs (respective library's ESTs > 90%) and singletons used to design the oligonucleotides. The two large *G. raimondii *libraries created from heterogeneous seedling and whole flower tissue are not illustrated. Click here for file
