# Supplementary figures and images for: Spotted cotton oligonucleotide microarrays for gene expression analysis
Source: BMC Genomics. 2007 Mar 27;8:81. doi: 10.1186/1471-2164-8-81 (PMC3225879; doi:10.1186/1471-2164-8-81)

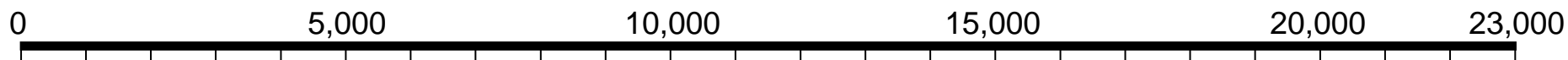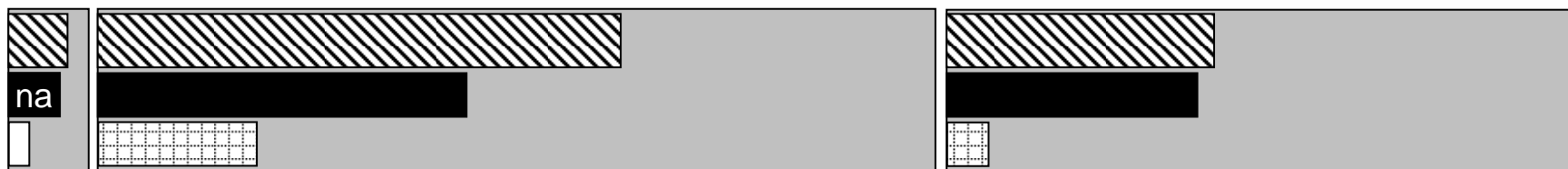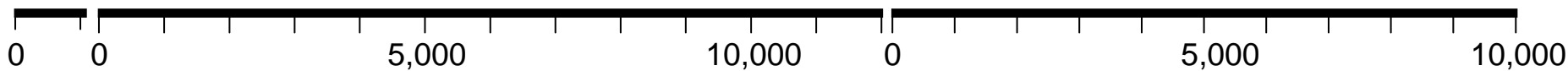

1,154  
1<sup>st</sup> oligo set

12,006 – 2<sup>nd</sup> oligo set

9,329 – 3<sup>rd</sup> oligo set

Supplement: Additional file 1 — Composition of the cotton oligonucleotide microarray. 22,789 oligonucleotides were designed from three separate sets of genic sequences from cotton (See Table 1). The grey boxes represent the total number of probes in each set. The hatched boxes indicate the number of probes with a putative Arabidopsis hit. The black boxes indicate the number of probes designed from singletons from their respective assemblies. The remaining boxes with dotted squares indicate the number of probes targeting transcription factors. [file 1471-2164-8-81-S1.pdf]

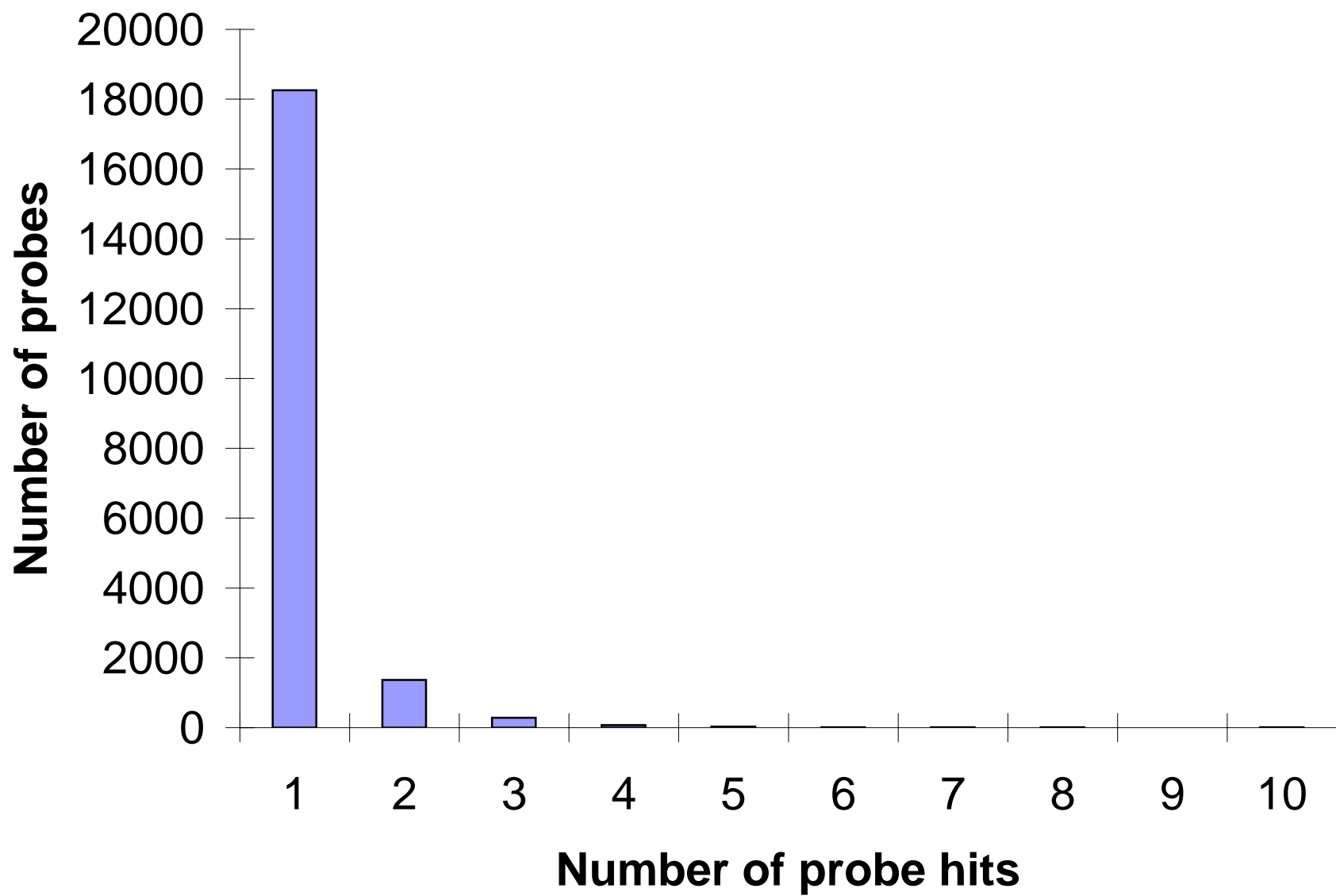

Supplement: Additional file 2 — Distribution of the number of matches of oligonucleotide probes to the Cotton Gene Index 8 (CGI8) assembly. All three sets were queried within the sequences of the CGI8 assembly and only a small number (1,773) of probes target (>93% percent identity) more than one CGI8 unigene indicating a potential cross-hybridization or an 'over-split' assembly for the targeted gene. [file 1471-2164-8-81-S2.pdf]
