# Supplementary material for: Spotted cotton oligonucleotide microarrays for gene expression analysis
Source: BMC Genomics. 2007 Mar 27;8:81. doi: 10.1186/1471-2164-8-81 (PMC3225879; doi:10.1186/1471-2164-8-81)
Supplement: Additional file 3 — Many of the oligonucleotides from the 2nd set were derived from contigs or singletons representing individual libraries (n = 7,319). Library totals reflect the contigs (respective library's ESTs > 90%) and singletons used to design the oligonucleotides. The two large G. raimondii libraries created from heterogeneous seedling and whole flower tissue are not illustrated. [file 1471-2164-8-81-S3.pdf]

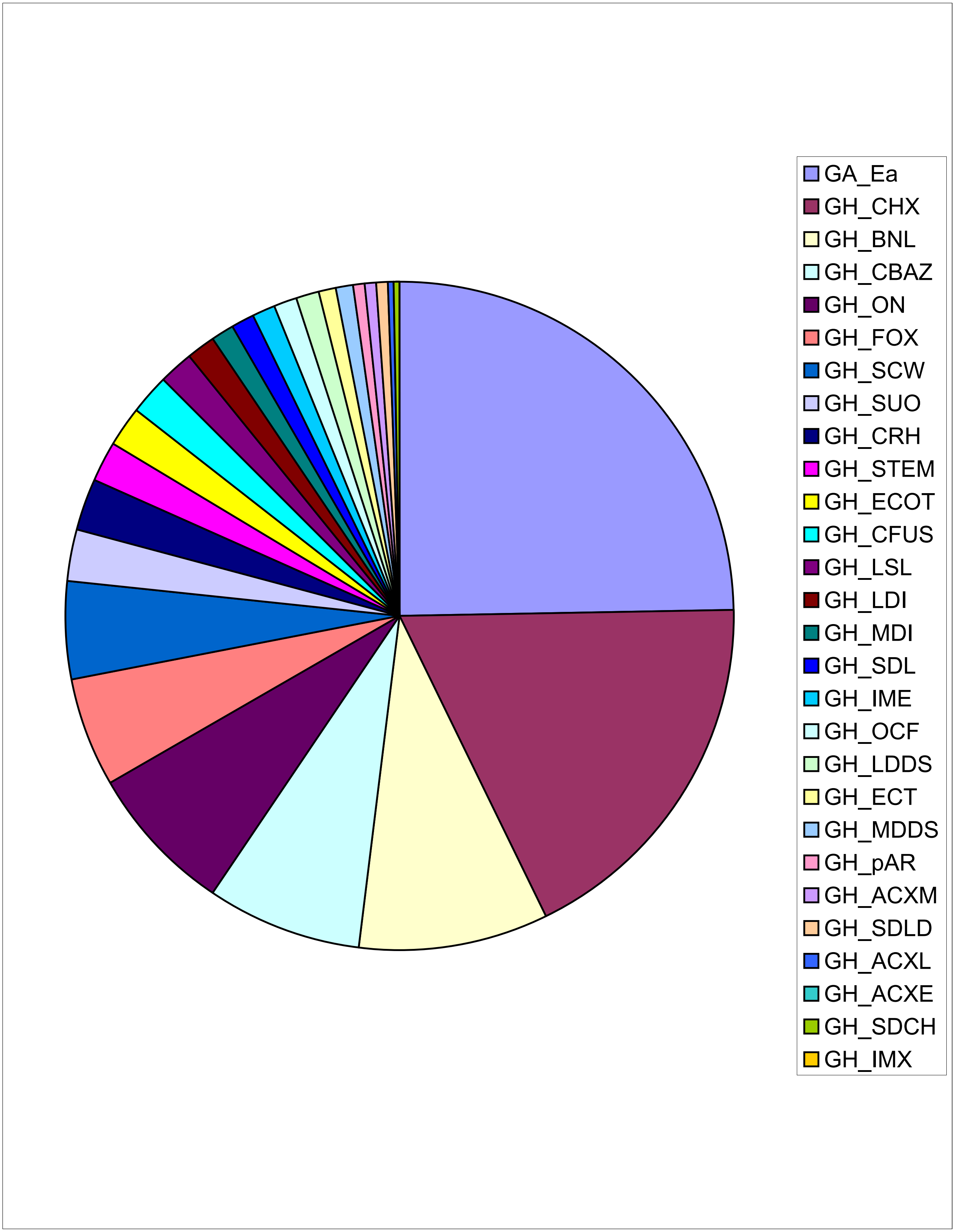
GA_Ea
GH_CHX
GH_BNL
GH_CBAZ
GH_ON
GH_FOX
GH_SCW
GH_SUO
GH_CRH
GH_STEM
GH_ECOT
GH_CFUS
GH_LSL
GH_LDI
GH_MDI
GH_SDL
GH_IME
GH_OCF
GH_LDDS
GH_ECT
GH_MDDS
GH_pAR
GH_ACXM
GH_SDLD
GH_ACXL
GH_ACXE
GH_SDCH
GH_IMX
